# Definition of Health 2.0 and Medicine 2.0: A Systematic Review

**DOI:** 10.2196/jmir.1350

**Published:** 2010-06-11

**Authors:** Tom H Van De Belt, Lucien JLPG Engelen, Sivera AA Berben, Lisette Schoonhoven

**Affiliations:** ^2^Scientific Institute for Quality of HealthcareRadboud University Nijmegen Medical CentreNijmegenNetherlands; ^1^Regional Emergency Healthcare NetworkRadboud University Nijmegen Medical CentreNijmegenNetherlands

**Keywords:** Health 2.0, Medicine 2.0, eHealth, Patient Empowerment, Professional Empowerment, Web 2.0, telemedicine

## Abstract

**Background:**

During the last decade, the Internet has become increasingly popular and is now an important part of our daily life. When new “Web 2.0” technologies are used in health care, the terms “Health 2.0" or "Medicine 2.0” may be used.

**Objective:**

The objective was to identify unique definitions of Health 2.0/Medicine 2.0 and recurrent topics within the definitions.

**Methods:**

A systematic literature review of electronic databases (PubMed, Scopus, CINAHL) and gray literature on the Internet using the search engines Google, Bing, and Yahoo was performed to find unique definitions of Health 2.0/Medicine 2.0. We assessed all literature, extracted unique definitions, and selected recurrent topics by using the constant comparison method.

**Results:**

We found a total of 1937 articles, 533 in scientific databases and 1404 in the gray literature. We selected 46 unique definitions for further analysis and identified 7 main topics.

**Conclusions:**

Health 2.0/Medicine 2.0 are still developing areas. Many articles concerning this subject were found, primarily on the Internet. However, there is still no general consensus regarding the definition of Health 2.0/Medicine 2.0. We hope that this study will contribute to building the concept of Health 2.0/Medicine 2.0 and facilitate discussion and further research.

## Introduction

During the last decade, the Internet has become increasingly popular and now forms an important part of our daily life [1]. In the Netherlands, the Internet is even more popular than traditional media like television, radio, and newspapers [2]. Furthermore, the impact of the Internet and other technological developments on health care is expected to increase [3,4]. Patients are using search engines like Google and Bing to find health related information. In Google, five percent of all searches are health related [5]. Patients can express their feelings on weblogs and online forums [3], and patients and professionals can use the Internet to improve communication and the sharing of information on websites such as Curetogether [6] and the Dutch website, Artsennet [7] for medical professionals. The use of Internet or Web technology in health care is called eHealth [1,8]. 

In 2004 the term “Web 2.0” was introduced. O’Reilly defined Web 2.0 as “a set of economic, social, and technology trends that collectively form the basis for the next generation of the Internet, a more mature, distinctive medium characterized by user participation, openness, and network effects” [9]. Although there are different definitions, most have several aspects in common. Hansen defined Web 2.0 as “a term which refers to improved communication and collaboration between people via social networking” [10]. According to both definitions, the main difference between Web 1.0 (the first generation of the Internet) and Web 2.0 is interaction [11]. Web 1.0 was mostly unidirectional, whereas Web 2.0 allows the user to add information or content to the Web, thus creating interaction. This is why the amount of “user-generated content” has increased enormously [12]. Practical examples of user-generated content are online communities where users can participate and share content. Examples are YouTube, Flickr, Facebook, and microblogging such as Twitter. Twitter, for example, improves communication and the sharing of information among health care professionals [13].

According to some critics, Web 2.0 is not a new generation of the Internet because it is still based on old technologies such as HTML, the predominant markup language. Therefore, the term Web 2.0 simply describes renewal or evolution of these older technologies or of the Internet itself [14,15]. Nonetheless, the term Web 2.0 seems to be widely used and accepted. The search engine Google recently found over 85,000,000 results for the search string “Web 2.0 or Web2.0.”

When Web 2.0 technologies are applied in health care, the term Health 2.0 may be used. [16,17]. Other authors use the term Medicine 2.0, which combines medicine and Web 2.0 [18]. There are many examples of Health 2.0/Medicine 2.0, such as the websites Patientslikeme [19] and Hello Health [20]. Recently, the Dutch minister of health awarded a grant to the website MijnZorgNet, which offers 23 virtual networks in which patients and their caregivers communicate. The networks are organized around specific patient categories. Successful examples that preceded the project are a digital in vitro fertilization (IVF) outpatient clinic [21,22] for couples receiving IVF treatment, and the website Parkinson Net [23] for people suffering from Parkinson’s disease. Both initiatives were started to enhance collaborative health care. Expected beneficial aspects of these projects were improved quality and efficiency of care [24]. Another concept that appears in the Health 2.0/Medicine 2.0 literature is “patient empowerment 2.0.” This has been described as “the active participation of the citizen in his or her health and care pathway with the use of information and communication technologies” [25]. It is assumed that Health 2.0/Medicine 2.0 leads to empowerment of the patient, as patients have easier access to health-related information and thereby have better understanding of choices that can be made.

According to Hughes [16], no relevant differences exist between Health 2.0 and Medicine 2.0. Eysenbach [18] agreed but stated, “If anything, Medicine 2.0 is the broader concept and umbrella term which includes consumer-directed ‘medicine’ or Health 2.0.” More and also more specific definitions of Health 2.0 and Medicine 2.0 exist [16,17]. However, these definitions seem to have evolved together with the increased use of the definitions and the different parties involved in Health 2.0 and Medicine 2.0. Ricciardi stated, “Everyone is trying to grasp what Health 2.0 exactly is” [26]. Does Health 2.0 refer to patients or to professionals or both? Does it focus on health care in general, or does it address specific aspects of health care like preventive or curative care, acute or chronic illness? Several authors concluded that there is no authoritative definition of the term yet, and Health 2.0 definitions and translations in practice remain murky and fragmented [27,28].

A clear definition is important for the development of new Health 2.0 or Medicine 2.0 initiatives and also for the comparability of new developments in research. Therefore, the aim of this study was to identify definitions of Health 2.0/Medicine 2.0 and to gain insight into recurrent topics associated with these labels.

## Methods

We performed a systematic literature study to find unique definitions of Health 2.0/Medicine 2.0 and identify and recurrent topics discussed in conjunction with these terms.

### Search Strategy

First, we searched the following electronic databases: PubMed, Scopus, and CINAHL. For each database, we searched all available years through September 2009. Since there was no relevant MeSH term available for Health 2.0 or Medicine 2.0, we used the following search terms: health 2.0, health2.0, health20, medicine 2.0, medicine2.0, medicine20, Web 2.0, Web2.0, Web20 (Table 1). We scanned the reference lists for relevant articles (the snowball method), contacted individual experts in the field, and inquired after relevant publications.

Second, we searched for gray literature on the Internet using the search engines Google, Bing, Yahoo, Mednar, and Scopus. Mednar and Scopus were used because they focus on scientific literature. Google, Bing, and Yahoo were used because these are the most widely used search engines [29,30]. We used the advanced search option, selected English as the preferred language, and turned the option for regional differences off. Based on earlier research [16], we expected a large number of results. Therefore we added a more specified search string query for Google, Yahoo, Bing, and Scopus (Table 2): “what is health 2.0,” “what is health2.0,” and “what is health20.” For Medicine 2.0 we used: “what is medicine 2.0,” “what is medicine20,” and “what is medicine20.” We studied the first 100 results in Google, Bing, and Yahoo as these search engines display results by relevance using a link analysis system or algorithms [31-33]. All searches in the gray literature were performed in November 2009.

### Inclusion Criteria

Subsequently, a combination of three of the authors (TB and LE and LS or SB) independently assessed the retrieved studies and gray literature for inclusion. Sources were included if a definition of Health 2.0 or Medicine 2.0 was identified. Disagreement over inclusion between the reviewers was resolved through discussion.

### Data Extraction

TH and LE independently assessed the included studies and gray literature and extracted unique definitions. A predesigned table was used to ensure standardized data extraction. For each definition we noted author, source, and year (Table 3). After completing the table, we used the constant comparison method to explore possible topics of Health 2.0 and Medicine 2.0 [34]. We independently analyzed the definitions and identified recurrent topics by using “coding.” Described by Strauss and Corbin, coding is an analytical process through which concepts are identified and dimensions are discovered in data [35]. The results are displayed in Table 4.

**Table 1:** Search strategy for scientific literature

Database/ Search Engine:	Search String:	Details	Hits	Relevant^a^	Included^b^
PubMed	“health 2.0” OR “health2.0” OR “health20” OR “medicine 2.0” OR “medicine2.0” OR “medicine20” OR “Web 2.0” OR “Web2.0” OR “Web20”		179	12	7
CINAHL	“health 2.0” OR “health2.0” OR “health20” OR “medicine 2.0” OR “medicine2.0” OR “medicine20” OR “Web 2.0” OR “Web2.0” OR “Web20”		199	4	0
Scopus	(TITLE-ABS-KEY(“health 2.0”) OR TITLE-ABS-KEY(“medicine 2.0”)) OR (TITLE-ABS-KEY(“health2.0”) OR TITLE-ABS-KEY(“medicine2.0”)) OR (TITLE-ABS-KEY(“health20”) OR TITLE-ABS-KEY(“medicine20”))		29	6	5
	(TITLE-ABS-KEY(“Web 2.0”) OR TITLE-ABS-KEY(“Web2.0”) OR TITLE-ABS-KEY(“Web20”)) AND (LIMIT-TO(SUBJAREA, “MEDI”) OR LIMIT-TO(SUBJAREA, “HEAL”) OR LIMIT-TO(SUBJAREA, “NURS”) OR LIMIT-TO(SUBJAREA, “MULT”))	Limited to subcategories: medicine, health professionals, nursing, multidisciplinary	126	3	2
Subtotal			533	25	14
Duplicates					5
Total			533	25	9

^a^ Relevant: number of relevant articles based on title, abstract, and keywords

^b^ Included: number of included articles based on full article

**Table 2:** Search strategy for gray literature

Database/ Search Engine	Search String:	Hits	Relevant^a^	Included^b^
Google	“health 2.0” OR “health2.0” OR “health20”	482000	28	13
	“medicine 2.0” OR “medicine2.0” OR “medicine20”	155000	24	16
	“what is health 2.0” OR “what is health 2.0” OR “what is health20”	99	29	25
	“what is medicine 2.0” OR “what is Medicine 2.0” OR “what is medicine 20”	33	14	14
Bing	“health 2.0” OR “health2.0” OR “health20”	328000	4	4
	“medicine 2.0” OR “medicine2.0” OR “medicine20”	62300	8	6
	“what is health 2.0” OR “what is health 2.0” OR “what is health20”	477	26	24
	“what is medicine 2.0” OR “what is medicine 2.0” OR “what is medicine 20”	31	12	11
Yahoo	“health 2.0” OR “health2.0” OR “health20”	466000	17	9
	“medicine 2.0” OR “medicine2.0” OR “medicine20”	45000	19	14
	“what is health 2.0” OR “what is health 2.0” OR “what is health20”	583	21	21
	“what is medicine 2.0” OR “what is medicine 2.0” OR “what is medicine 20”	121	14	12
Mednar	“health 2.0” OR “health2.0” OR “health20”	329	27	10
	“medicine 2.0” OR “medicine2.0” OR “medicine20”	12	13	5
Scopus	TITLE-ABS-KEY(“what is health 2.0”) OR TITLE- ABS-KEY(“what is health2.0”) OR TITLE-ABS-KEY(“what is health20”)	23	3	0
	TITLE-ABS-KEY(“what is medicine 2.0”) OR TITLE- ABS-KEY(“what is medicine2.0”) OR TITLE-ABS-KEY(“what is medicine20”)	0	0	0
Subtotal		1540008	262	184
Duplicates				149
Total				35

^a^ Relevant: number of relevant articles based on title, abstract, and keywords in first 100 results

^b^ Included: number of included articles based on full article

## Results

We scanned a total of 1937 articles, 533 found in scientific databases and 1404 in the gray literature (Tables 1 and 2). We selected 287 articles, 25 peer reviewed articles, and 262 non-scientific articles for further analysis. After selection and removing duplicates, we distinguished 46 unique definitions of Health 2.0 or Medicine 2.0 in 44 articles (Table 3). The length of the definitions varied from 7 to 105 words. We found 42 definitions describing Health 2.0 [3,15-18,25-27,36-69] and two definitions describing Medicine 2.0 [70,71]. Of the 44 articles included, 8 included definitions of both Health 2.0 and Medicine 2.0 [16-18,40,50,52,55,65]. From these 46 definitions, we identified 7 main recurrent topics: patients, Web 2.0/technology, professionals, social networking, change of health care, collaboration, and health information/content (Table 4). In the following paragraphs we describe these recurrent topics from these definitions in more depth.

### Patients and Consumers

The first main topic was “patients” or “consumers of health care,” which was found in 35 definitions. Of these, 12 included mention of either increased participation or empowerment of patients. The following terms or phrases were identified: increased consumer/patient participation [18,27,49,50,58], patients can actively participate [63], and participatory [42,45], patient empowerment or consumer empowerment [41,49,59,62]. The other 23 mentioned only patient or consumer involvement and not the effects.

### Web 2.0/Technology

The second main topic that appeared in 32 definitions from 30 articles was “Web 2.0” or “technology.” Terms varied from “Web 2.0” [3,15,17,36,43,44,46,52,55,57,58,60,62,67,70], to “Web 2.0 technology” [18,27,40,41,50,66,68], “technology” [25,39,62-64], “software” [42,51], “Web (based) tools” [69,71], and “ICT (information and communication technology)” [37]. Web 2.0 was seen as the total of available technologies that stakeholders could use for communication and for sharing information. One definition mentioned “mashing” of Web 2.0 concepts and tools [43]. “Mashing” was seen as combining two or more Web 2.0 sources to create a new one. Other definitions indicated that the concept of Health 2.0 originated from a combination of the concepts “health” and “Web 2.0” [17,40].

### Professionals

The third topic that was identified concerns “professionals” or “caregivers,” and was found in 26 definitions. Of the 46 included definitions, five mentioned increased participation or empowerment of professionals. The following terms were found: “professional empowerment” [49,52,59], “empowerment of the individual” [48], and “empowerment of the user” [3].

Besides patients and professionals, other stakeholders were mentioned. However, they were mentioned less frequently and therefore not included in Table 4 as individual topics. The following stakeholders were mentioned: payers or providers [36,44,52,61], medical and health science students [27,52], biomedical researchers [18,44,49,50,52,71], entrepreneurs [62,65], and government [44]. Other authors were less specific with regard to stakeholders. They included “all stakeholders” [38] or “others” [43,51,57,66].

### Social Networking

The fourth topic, the emergence of online communities and social networking, was reflected in 22 definitions. This was described using different terminology. Definitions referred to “online communities” [42,47,48,51,52,58,66], “social communities” [44], “networks” [71], whereas others referred to “online social networks” or “social networking” [18,26,36,43,50,59], “social interaction” [36], “interactive environments” [58], or “intelligent interaction” [63]. Other definitions focused more on technology: the terms used were “social media tools” [60], “social media,” or “social software” [38,46,56,59,69].

Two authors mentioned “transparency” or “openness” [18,49]. An additional 2 definitions suggested that “sharing” or “online sharing” of medical information was part of Health 2.0 or Medicine 2.0 [45,65].

### Change of Health Care

Fifth, we found that change of health care was described by 15 definitions. According to the definitions, Health 2.0 means change of health care: “a whole new way of involving consumers in the health care system” [64], “next generation of health care services” [67], “new and better health system” [18], “new concept of health care” [52], “all constituent focus on health care value and on improving safety, efficiency and quality of health care” [61], “shaping health care with Web 2.0 tools” [17], and “new wave of innovation” [62]. Change was described differently: “reshaping health care”[17,42], “ever changing” [66], “continually evolving cycle” [49], “evolution of technology and medical industry” [36], “evolution of health care” [41]. Change was also described as “revolutionary” [55], while another author stated, “we should be careful not to assume that a revolution has occurred in health care” [27].

We also found one author who referred to “user generated health care” [25].

### Collaboration

The sixth topic, mentioned in 14 definitions, was collaboration. In the Health 2.0 era, patients will actively contribute to their own care process. Collaboration between professionals and patients may improve. Terms varied from “collaboration” [18,36,43,49,51,59,66,69], “collaboratively” [27], “collaborate” [52,71], “collaborative practices” [16], and “collaborate and share knowledge” [70] to “working together” [39].

There were also other aspects described with regard to the relationship among stakeholders. Patients would transform their role in health care [26] and would be on the same level of playing field as other stakeholders [38]. A role change of patients and professionals was also indicated. For example, the following phrase was used: “doctor and patient positioned together” [37]. Patients were described as “active contributors” [55], “active and responsible partners” [25], or “active partners” [42]. Another author mentioned “integration of patients and stakeholders” [45].

### Health Information or Content

Seventh and last, there was mention of health information or content in 14 definitions. Terms varied from “information,” “health information,” or “medical information” [27,36,37,42,45,48,53,63,65] to “content” [47], “data” [26,44,71], and “user owned content” [58].

**Table 3:** Definitions of Health 2.0 and Medicine 2.0

Author, Source, and Whether Found in Scientific Literature^a^ or Gray Literature^b^	Year of Publication	Definition
Aller RD et al [36] (Gray)	2007	The term, boiled down to its most basic definition, refers to the evolution of technologies and the medical industry itself to create the next generation of health care for consumers, providers, and payers alike. The term is a take on Web 2.0, which refers to the evolution of the Internet from a tool used essentially for information gathering to one used for collaboration and social interaction.
Bos L et al [25] (Scientific)	2008	Health 2.0 is user generated Health care. What is foreseen is that the self-care information tool of the future will be a combination between the patient's observation record and the Internet, with the doctor and the patient positioned together at the intersection but not having to pay attention to the technology.
Bos L et al [37] (Scientific)	2008	Health 2.0 defines the combination of health data and health information with (patient) experience through the use of ICT, enabling the citizen to become an active and responsible partner in his/her own health and care pathway.
Bourre N [38] (Gray)	2009	Social media and conversations related to health care, where all stakeholders are on the same level of the playing field.
Castilla V [39] (Gray)	Unknown	Medicine 2.0 is about realizing the potential of today's technology in health care. Medicine 2.0 is about working together. Medicine 2.0 is about getting closer to colleagues and patients.
Conn J [15] (Scientific)	2007	The health care derivate of the far more ubiquitous "Web 2.0."
Doherty I [27] (Scientific)	2008	Web 2.0 Technologies provide members of the health community–health professionals, health consumers, health carers, and medical and medical and health science students–with new and innovative ways to create, disseminate, and share information both individually and collaboratively. This phenomenon has been termed Health 2.0. There is no authoritative definition of the term yet. Health 2.0 is in its infancy and we should be careful not to assume that a revolution has occurred in health care as a result of these new technologies and their various affordances.
Dolan F [40] (Gray)	2007	Health 2.0 is the application of Web 2.0 technologies in the area of health, while Medicine 2.0 is the use of Web 2.0 technologies in the area of medicine.
Dubay A [41] (Gray)	2007	Health 2.0 is the evolution of health care as a result of consumer empowerment. Its definition ranges from “applied Web 2.0 technology to health care” to “the next generation health care delivery.”
Eysenbach G [18] (Scientific)	2008	Medicine 2.0 applications, services, and tools are Web-based services for health care consumers, caregivers, patients, health professionals, and biomedical researchers, that use Web 2.0 technologies and/or semantic web and virtual-reality tools, to enable and facilitate specifically social networking, participation, apomediation, collaboration, and openness within and between these user groups. Or in broader concept: medicine also stands for a new and better health system, which emphasizes collaboration, participation, apomediation, and openness, as opposed to the traditional, hierarchical, closed structures within health care and medicine. Medicine 2.0 is the broader concept and umbrella term, which includes consumer-directed "medicine" of Health 2.0.
Eytan T [42] (Gray)	2008	Health 2.0 is participatory health care. Enabled by information, software, and community that we collect or create, we the patients can be effective partners in our own health care, and we the people can participate in reshaping the health system itself.
Facebook Health 2.0 Group [43] (Gray)	2007	Health 2.0 is the mashing of Web 2.0 concepts and tools to health care industry, including social networking to promote better collaboration between patients, their caregivers, medical professionals, and others involved in the health care industry.
Flock, B [44] (Gray)	2008	Health 2.0: Expand initial Health care 2.0 concept (Web 2.0 features to health care; ratings, search, social communities, and consumer tools) to include entire Health ecosystem (payers, providers, employers, consumers, life sciences entities, government: anyone who can contribute meaningful data.)
Furst I [45] (Gray)	2008	Health 2.0 is participatory health care characterized by the ability to rapidly share, classify, and summarize individual health information with the goals of improving health care systems, experiences, and outcomes via integration of patients and stakeholders.
Gavgani VZ et al [70] (Scientific)	2008	Medicine 2.0 is the latest approach to ensure better health system and well-being of the humanity, in other words, “health for all,” and a healthy community. The development of Medicine 2.0 grossly depends on the application of Web 2.0 sciences.
Goel V [46] (Gray)	Unknown	Health 2.0 is the use of social media and other technologies to improve communication in health care. These platforms may be used to connect patients with patients, doctors with other professionals, or patients with doctors. The Health 2.0 movement is about enhancing communication to improve the focus and results of the health system on the patients it serves.
Goreman J et al [47] (Gray)	2008	Health 2.0: The combination of content and community.
Halper R [48] (Gray)	2007	The empowerment of the individual to have access to detailed objective health care information primarily, though not exclusively, using search engine sites and like-minded communities of patients and physicians.
Hawker M [49] (Gray)	2008	Health 2.0 is a continually evolving cycle of health care innovation enabled by the empowerment of the public, patients, health care providers and suppliers, and researchers through increased collaboration, participation, apomediation, feedback and transparency of value-enabled health care interactions.
Healthcaremanagementblog [50] (Gray)	2008	Health 2.0 aka Medicine 2.0 aka eHealth, can be broadly defined as “applications, services, and tools are Web-based services for health care consumers, caregivers, patients, health professionals, and biomedical researchers, that use Web 2.0 technologies as well as semantic web and virtual reality tools, to enable and facilitate specifically social networking, participation, apomediation, collaboration, and openness within and between these user groups.”
Holt M [51] (Gray)	2007	The use of social software and lightweight tools to promote collaboration between patients, their caregivers, medical professionals, and other stakeholders in health.
Hughes B [16] (Scientific)	2008	Health 2.0 and Medicine 2.0 were found to be very similar and subsume five major salient topics: (1) the participants involved (doctors, patients, etc); (2) its impact on both traditional and collaborative practices in medicine; (3) its ability to provide personalized health care; (4) its ability to promote ongoing medical education; (5) its associated method- and tool-related issues, such as potential inaccuracy in end user-generated content. Difference Health 2.0 and Medicine 2.0 with eHealth, the key distinctions are made by the collaborative nature of Health 2.0 and Medicine 2.0.
Jessen W [52] (Gray)	2008	Medicine 2.0 is the science of maintaining and/or restoring human health through the study, diagnosis, and treatment of patients utilizing Web 2.0 Internet-based services, including Web-based community sites, blogs, wikis, social bookmarking, folksonomies (tagging) and Really Simple Syndication (RSS), to collaborate, exchange information, and share knowledge. Physicians, nurses, medical students, and health researchers who consume Web media can actively participate in the creation and distribution of content, helping to customize information and technology for their own purposes.
		Health 2.0, a new concept of health care, also utilizes Web 2.0 Internet-based services but is focused on health care value (meaning outcome/price). Patients, physicians, providers, and payers use competition at the medical condition level over the full cycle of care as a catalyst for improving safety, efficiency, and quality of health care delivery. The goal of both of these movements is the delivery of optimal medical outcomes though individualized care.
Levine C [53] (Gray)	2009	Health 2.0 = a noun that describes user-generated health care content. Spurred by sites like YouTube, Facebook, and Wikipedia, millions are logging on to contribute information and opinions on everything from medications, health professionals, treatment options, side effects, flu pandemics, and best drug practices.
Mesko B [17] (Gray)	2007	Medicine 2.0 = Web 2.0 + medicine (focusing on doctor-patient communication and technologies).
		Health 2.0 = Web 2.0 + health care (focusing on shaping health care with Web 2.0 tools and concepts).
Maun C [54] (Gray)	2009	Health 2.0 can be broadly defined as interactive applications, services, and tools that are Web-based services for health care consumers, caregivers, patients, and health professionals.
Moturu ST et al [55] (Scientific)	2008	Like the Web 2.0 revolution changed the user from a passive consumer to an active contributor, a similar metamorphosis being termed as Health 2.0 or Medicine 2.0 would extend the role of information seeking users to include dissemination of experiences and acquired knowledge.
Rampy A [56] (Gray)	2008	Health 2.0 = the merging of social media into health care.
Randeree E [3] (Scientific)	2008	Health care 2.0 can be defined as a network of (Web 2.0) applications and services that empower the user and are delivered through the web as a platform.
Ricciardi L [26] (Gray)	2008	Its grassroots push through which patients are using social networks and other tools to generate their own health data and transform their role vis a vis the health care system. Quite honestly, everyone is still trying to figure out exactly what Health 2.0 is.
Richlovsky P [58] (Gray)	2007	Basically, Health 2.0 is a takeoff of Web 2.0, and it alludes to health websites that incorporate Web 2.0 principles of encouraging user-generated and user-owned content, participation, and community-building in rich, interactive environments.
RN Central [57] (Gray)	2008	Health 2.0 embraces the idea of bringing health care into the community of medical professionals, patients, and those in the health care industry together with technology and the Internet to provide the best possible health care environment.
Sarashon-Kahn J [59] (Gray)	2007	Social media on the Internet are empowering, engaging, and educating consumers and providers in health care. This movement, known as Health 2.0, can be defined as: The use of social software and its ability to promote collaboration between patients, their caregivers, medical professionals, and other stakeholders in health.
Sharp J [60] (Gray)	2009	Health 2.0 evolved more recently and focuses on Web 2.0 tools, especially social media tools, and their use in health care.
Shreeve S [61] (Gray)	2007	Health 2.0: New concept of health care wherein all the constituents (patients, physicians, providers, and payers) focus on health care value (outcomes/price) and use disruptive innovation as the catalyst for increasing access, decreasing cost, and improving the quality of health care.
Spoetnik L [71] (Gray)	2009	Medicine 2.0 is the use of a specific set of Web tools (blogs podcasts, tagging, search, wikis, etc) by actors in health care, including doctors, patients, and scientists, using principles of open source and generation of content by users and the power of networks in order to personalize health care, collaborate, and promote health education.
Stoakes U [62] (Gray)	2008	Health 2.0: A new wave of innovation in health care as a result of changing trends in technology, consumer empowerment, and growing entrepreneurialism at a time when the health care system is spiraling out of control. These converging trends have created an environment for entrepreneurs, start-up companies, innovative thinkers, health professionals, and consumers to rethink how to solve today’s biggest health care challenges. Health 2.0 is about coming up with new ideas and rethinking what’s possible.
Susheel-Ommen J [3] (Gray)	2007	Health 2.0 derives its definition from the definition of Web 2.0, where the technologies used allowed intelligent interaction between the users and the deployed solutions. Currently available technologies allow users to actively participate and contribute to the information that is front-ended using Web interfaces.
Tenderich A [64] (Gray)	2009	It’s both an explosion in new Web-based personal health technologies and a whole new way of involving consumers in the health care system.
Torrey T [65] (Gray)	2008	Medicine 2.0 or Health 2.0 are terms used to describe the massive Internet-sharing of health and medical information among everyone with interest, from health and medical professionals, to patients, to caregivers, to the businesses (pharmaceutical manufacturers, health insurance) which support them. The two terms, Medicine 2.0 and Health 2.0, are often used interchangeably. However, there is a distinction. Medicine 2.0 usually refers to the science of medicine and the practice of treating or curing patients. Health 2.0 is focused on the business of health in general including the delivery, the quality, the safety, and the cost or efficiency of the people, a practice, or facility.
Venn D [66] (Gray)	2008	Health 2.0 is an emerging concept of health care that uses Web 2.0 technologies to promote collaboration between patients, physicians, health care professionals, and other members of the health community. Its application is ever-changing, and the evidence for its effectiveness is still raw, but there’s a lot of potential for this type of new technology to improve mental health education and mental health care.
Weisbaum W [67] **(**Gray)	2007	Health 2.0 is the use of movement to harness the technology of Web 2.0 for the delivery of the next generation of health care services.
Williams P [68] (Gray)	Unknown	Health 2.0 is the use of Web technology to deliver consumer-driven health services. It uses the same Web 2.0 technology that drives the successful Internet services such as Ebay, Facebook, Expedia, and Amazon.
Wright-Mark S [69] (Gray)	2008	Health 2.0 is a new concept of health care that employs social software and other Web-based tools to promote collaboration between patients, their caregivers, medical professionals, and other stakeholders in health care to create a better, more knowledgeable and cost effective environment for better well-being.

^a^ Located with search of the following databases: PubMed, Scopus, and CINAHL

^b^ Located using the search engines Google, Bing, Yahoo, Mednar, and Scopus

**Table 4:** Recurrent topics of Health 2.0 and Medicine 2.0

Author and Definition of Health 2.0 (H2) and/or Medicine 2.0 (M2)			Topics						
Author	H2	M2	Patients and Consumers	Web 2.0	Professionals	Social Networking	Change	Collaboration	Health Information or Content
Aller RD et al [36]	*		*	*	*	*	*	*	*
Bos L et al [25]	*		*	*	*				
Bos L et al [37]	*		*	*					*
Bourre N [38]	*					*			
Castilla V [39]	*		*	*				*	*
Conn J [15]	*			*					
Doherty I. [27]	*		*	*	*		*	*	*
Dolan F [40]	*	*		*					
Dubay A [41]	*		*	*			*		
Eysenbach G [18]	*	*	*	*	*	*	*	*	
Eytan T [42]	*		*	*		*	*		*
Facebook Health 2.0 Group [43]	*		*	*	*	*		*	
Flock, B [44]	*		*	*	*	*			*
Furst I [45]	*		*		*				*
Gavgani VZ et al [70]		*	*	*					
Goel V [46]	*		*	*	*	*			
Goreman J et al [47]	*					*			*
Halper R [48]	*		*		*	*			*
Hawker M [49]	*		*		*		*	*	
Health caremanagementblog [50]	*	*	*	*	*	*		*	
Holt M [51]	*		*	*	*	*		*	
Hughes B [16]	*	*	*		*			*	
Jessen W [52]		*		*	*	*		*	
	*		*	*	*		*		
Levine C [53]	*		*						*
Mesko B [17]	*		*	*	*		*		
		*	*	*	*		*		
Maun C [54]	*		*		*				
Moturu ST et al [55]	*	*					*		
Rampy A [50]	*					*			
Randeree E [3]	*		*	*					
Ricciardi L [26]	*		*			*			
Richlovsky P [58]	*			*		*			*
RN Central [57]	*		*	*	*				
Sarashon-Kahn J [59]	*		*		*	*		*	
Sharp J [60]	*			*		*			
Shreeve S [61]	*		*		*		*		
Spoetnik L [71]		*	*	*	*	*		*	*
Stoakes U [62]	*		*	*	*		*		
Susheel-Ommen J [63]	*		*	*		*			*
Tenderich, A [64]	*		*	*			*		
Torrey T [65]	*	*	*		*				*
Venn D [66]	*		*	*	*		*	*	
Weisbaum W [67]	*			*			*		
Williams P [68]	*			*					
Wright-Mark S [69]	*		*	*	*	*		*	

## Discussion

This literature search resulted in 46 unique definitions in 44 articles of Health 2.0/Medicine 2.0 in scientific databases and gray literature on the Internet. We distinguished seven recurrent topics: Web 2.0/technology, patients, professionals, social networking, health information/content, collaboration, and change of health care. 

This study showed that the use of the terminology differed among the definitions mentioned in literature. The term Health 2.0 was included in 42 definitions, 10 definitions mentioned Medicine 2.0, and 6 definitions described Health 2.0 and Medicine 2.0 as equal. There were 36 definitions that only mentioned the term Health 2.0, and only 4 definitions that described Medicine 2.0. Although some authors indicated that little or no differences existed between the two terms [16,18,27,55], others saw differences, for example that Medicine 2.0 is focused on the relation between professionals and patients whereas Health 2.0 is focused on health care in general [17,52,65]. As most definitions described Health 2.0, this term may be more widely used and accepted than Medicine 2.0.

Overall, we found that the term Web 2.0 was mentioned often: 33 authors used the term directly in the definition, which suggests that they accepted this concept. However, others state that Web 2.0 does not exist at all [72]. Authors’ interpretations of the meaning of Web 2.0 influenced their definitions of Health 2.0/Medicine 2.0 profoundly. We generally distinguished two meanings of Web 2.0. The first meaning is that Web 2.0 is a set or “mashing” (ie, a combination) of technological developments [51,58]. The second meaning is that Web 2.0 is a new generation of the Internet where interaction is important, with more user-generated content that empowers people. In this interpretation, technology, or the mashing of different technologies, is only a tool, and Web 2.0 is more than technology. These meanings result in different definitions of Health 2.0/Medicine 2.0. A number of definitions referred to the technological developments embedded in health care, whereas other definitions stated that Health 2.0/Medicine 2.0 is a new generation of health care. We believe Web 2.0 is a facilitator for Health 2.0/Medicine 2.0, but not a necessity. Indeed, patients can still access health related information without Web 2.0; for example, a patient can go to a library and become well-informed without Web 2.0 technology. However, this would be far more difficult than becoming well-informed through the use of Web 2.0 technology. Second, the topic of stakeholders reflects who the main players are in the field of Health 2.0/Medicine 2.0. The two main stakeholders we distinguished were patients or consumers, mentioned in 35 definitions, and professionals or caregivers, mentioned in 26 definitions. Interestingly, other stakeholders such as payers of health care, scientists, students, and entrepreneurs were mentioned less frequently, whereas the government was only mentioned once. This is particularly interesting as the government has great influence on health care and changes in health care. Apparently the government is not yet an active party in the development of Health 2.0/Medicine 2.0.

Also interesting was that most definitions focused on the relation between patients and professionals. With Health 2.0/Medicine 2.0, patients and professionals were seen to collaborate, with patients transforming their role in health care using social networks and access to health information. Moreover, other relationships might also change; for example, the appearance of online communities could change the relationship between health professionals and specific groups of patients. This has been termed collaborative health care [18].

Finally, it is expected that Health 2.0/Medicine 2.0 will lead to change of health care. Expectations concerning the speed of this change ranged from a “gradual shift” [27], an “ever changing” [66] or “continuous interactive process” [49] to “revolution” [55]. However, we advise caution in assuming that a revolution has taken place [27]. It may be that communication, information exchange, and patients’ contribution to his or her care has improved or accelerated, but according to Engelen [8], no fundamental changes in health care have yet occurred.

Authors of a Health 2.0/Medicine 2.0 definition generally seemed to approach the definition from their own perspective. For example, patients or patient federations saw patients as the main stakeholder and focused on empowerment of the patient. That is, definitions may be influenced by different stakeholders’ agendas. Therefore, it is important for future Health 2.0/Medicine 2.0 researchers to incorporate all stakeholders and thereby include all possible views and perspectives.

### Limitations

Our study has some limitations. First, we found 46 unique definitions, mostly in the gray literature, using the Internet. Only 9 definitions were found in peer-reviewed articles in the scientific literature. This can be explained by the fact that Health 2.0/Medicine 2.0 is a relatively new concept and is still developing. However, it is important to realize there is no evidence-based method available to determine the quality of online content yet. Consequently, proper assessment of the value of the definitions we found was not possible.

Second, it appeared that searches using Google, Bing, and Yahoo showed many results. Although these search engines displayed results by relevance using algorithms and ranking systems, we may have missed unique definitions as we only studied the first 100 results.

Finally, the exact way search engines display results remains unclear. The process can be seen as a black box. As a result, reproduction of searches is far from optimal, as the results literally change every second. Therefore, one might question the suitability of these search engines for scientific research. However, by combining the results of Google, Bing, and Yahoo and using four search queries, we believe we found the majority of all relevant definitions in the gray literature.

### Conclusion

Health 2.0/Medicine 2.0 is still a developing concept. Our study identified 46 unique definitions of Health 2.0 and Medicine 2.0 with seven recurrent topics: Web 2.0/technology, patients, professionals, social networking, health information/content, collaboration, and change of health care. There is no general consensus of the definition of Health 2.0/Medicine 2.0 yet. We hope that this study will contribute to building the concept of Health 2.0/Medicine 2.0 and facilitate future discussion and research to achieve a clear conceptual framework.

## Abbreviations

ICT: information and communication technology
IVF: in vitro fertilization
